# Bridging gaps in veterinary care: restructuring a community clinic for cultural inclusion in Phoenix, Arizona

**DOI:** 10.3389/fvets.2025.1595312

**Published:** 2025-10-24

**Authors:** Champayne Danae Master, Marjorie Robin Vincent, Bridgid Ann Twomey, Claudia Vazquez, Jyothi Vinnakota Robertson

**Affiliations:** ^1^Arizona Animal Welfare League, Phoenix, AZ, United States; ^2^JVR Strategies, Belmont, CA, United States

**Keywords:** promotoras, access to veterinary care, Latino communities, barriers, community clinic, needs assessment

## Abstract

This case study examines the Arizona Animal Welfare League's (AAWL) efforts to bridge gaps in veterinary care access for Latino communities in Phoenix, AZ, with strategic guidance from JVR Strategies supported by Maddie's Fund. To better understand the specific needs, barriers, and preferences for pet health services, bilingual community needs assessments were conducted through partnerships with PetSmart Charities, Community Alliance Consulting, LLC, the Alliance for Companion Animals Coalition, and the Institute for Human-Animal Connection (IHAC). Insights gained from these needs assessments shaped the restructuring of AAWL's public clinic, leading to the development of a Saturday-only clinic model, bilingual services, outreach, and off-site clinics in targeted zip codes. While the transition posed operational challenges, early results demonstrated increased patient volume, expanded access to care, and created stronger connections with the community. This restructuring created space to listen more deeply to residents through informal conversations and relationship-building in the neighborhoods AAWL sought to serve. Community members consistently pointed to promotoras—local community health workers—as the trusted individuals they already turn to for information, resources, and guidance. Recognizing this, AAWL began building relationships with promotoras, who served as trusted connectors linking community members to AAWL and the veterinary services available to them. This case study examines the organization's process from the initial assessments in 2022 through the clinic's transition to a new model in March 2024, with ongoing adjustments to the model based on community feedback through July 2024. It offers insights into the importance of inclusive needs assessments, community partnerships, and culturally relevant service delivery. These findings provide a framework for organizations aiming to develop community-based veterinary care programs that are both equitable and sustainable.

## 1 Introduction

Community-based veterinary medicine, which refers to clinic models tailored to underserved populations, plays a critical role in addressing barriers to accessing veterinary care. Various models, such as veterinary teaching hospitals, mobile units, and low-cost clinics, have been developed to improve accessibility. Persistent challenges, including financial constraints, geographic limitations, cultural differences, and operational inefficiencies, continue to impede progress (1). These barriers force pet owners to navigate obstacles and endure hardships before securing veterinary services (2). A systematic review revealed significant barriers to veterinary care, including cost, accessibility, and communication issues (3). A more recent scoping review expanded on this by highlighting financial, geographic, and systemic barriers and proposed a more inclusive, equity-focused definition for access to care (4). Both reviews emphasized the need for more research into community-based veterinary programs and the development of new models for accessible care. The repercussions of barriers to care are widespread and may contribute to reduced trust and engagement with veterinary services, particularly in communities historically excluded from care systems (5). When pets are provided care, the incidence of preventable diseases in the population decreases, including zoonotic diseases that risk public health (6). Furthermore, the human-animal bond may weaken as a result of a lack of access to veterinary care, leading to increased stress and potential economic burdens on families (7). On a broader scale, the community can experience deteriorating public health and increased strain on already overburdened animal shelters, which struggle to accommodate the influx of surrendered animals due to preventable health issues (8).

The Arizona Animal Welfare League (AAWL) sought to bridge gaps in veterinary care in the predominantly Latino communities of Central City and Maryvale using community-based initiatives. Founded in 1971, AAWL cares for homeless pets, partners with rural and local partners to reduce euthanasia rates, and expands access to low-cost veterinary services to the public. To address the significant unmet needs in its immediate area, AAWL began restructuring its existing public clinic model while also exploring ways to bring services directly into the neighborhoods through off-site clinics and community partnerships. The development of a clinic model that better aligned with the community's cultural, linguistic, and socioeconomic realities was necessary to successfully provide care in these communities. With this goal in mind, AAWL revamped its pre-existing full-service clinic into what is now known as the Community Vet Clinic (CVC), a high-volume wellness clinic tailored to the needs of the community, and added monthly offsite preventative care clinics in the targeted communities.

Each community faces unique social, cultural, and structural dynamics that could inform an approach to providing accessible veterinary care. Many studies have documented the impact of low-cost spay/neuter services, but there has been less focus on low-cost wellness and prevention-such as vaccinations, flea/tick and heartworm testing, and related medications (9). This paper documents AAWL's approach to identifying and addressing gaps in care, including its assessment of community needs, clinic restructuring, and early implementation outcomes. By offering insights into the practical implications of a community-engaged approach, the authors highlight considerations that may inform similar efforts in other communities- particularly in areas with significant cultural and linguistic diversity. Aspects of AAWL's process, including bilingual outreach, flexible clinic models, listening to the community, using community input to inform decision-making and information delivery, and trust-building, may be adapted to align with the needs of other populations facing barriers to veterinary care.

## 2 Case description

### 2.1 Community context

Phoenix, Arizona, is home to approximately 1.6 million people, with 41% identifying as Hispanic or Latino (10). Central City Phoenix and Maryvale communities are among the most economically challenged areas in the city and face some of the greatest barriers to veterinary care.

Central City, which encompasses downtown Phoenix and its surrounding residential neighborhoods (11) has a poverty rate of 29.6%, nearly double the county-wide average of 16.5%. The median household income is $34,901, less than half of Maricopa County's $76,697, and unemployment in the area stands at 8.9%, nearly double the national average, reflecting limited job opportunities and economic instability. Only 26.9% of Central City residents have a bachelor's degree or higher, significantly lower than the 41.2% countywide rate. Research has shown that educational attainment is closely linked to earning potential, with lower levels of education often leading to fewer job prospects, lower wages, and reduced financial stability (12). These economic conditions leave many residents with little discretionary income, making it difficult to afford non-essential expenses, including veterinary care.

Maryvale, located west of downtown, faces similar economic hardships. The poverty rate is 19% and the median household income is $49,429, significantly below the county median. Unemployment in Maryvale is 10.6%, further limiting economic mobility. Educational attainment is also notably lower, with only 13% of residents holding a bachelor's degree or higher, making it more difficult for individuals to access higher-paying jobs and financial stability. A comparison of key socioeconomic and demographic indicators for Central City, Maryvale, and Maricopa County is presented in Table 1, highlighting disparities in income, education, poverty rates, and language diversity.

**Table 1:** Socioeconomic and demographic comparisons of Central City, Maryvale, and Maricopa County (11, 30–32).

**Area**	**% of households below federal poverty line**	**% bachelor's degree or higher**	**Median household income**	**% of Latino residents**	**% Speaking a language other than english at home**	**% of Unemployed residents**
Central City Community	29.6%	26.9%	$34,901	54.4%	49.0%	8.9%
Maryvale Community	19.0%	13.0%	$49,429	77.6%	64.8%	10.6%
Maricopa County	16.5%	41.2%	$76,697	30.5%	26.6%	8.0%

Cultural and linguistic barriers further limit access to veterinary services in both Central City and Maryvale, where large portions of the population primarily speak Spanish at home. In Central City, 54.4% of residents identify as Latino, and 49% speak a language other than English at home, primarily Spanish. In Maryvale, these figures are even higher, with 77.6% identifying as Latino and 64.8% speaking a language other than English at home. Limited English proficiency can create communication challenges in both medical (13) and veterinary settings (14), discouraging residents from seeking care and reducing their ability to fully understand treatment options, costs, and preventive care recommendations.

Informal feedback gathered through community conversations and outreach by the organization also highlighted cultural barriers such as limited availability of bilingual staff, unfamiliar or clinical-feeling environments, and communication styles that felt disconnected or impersonal to some residents. These perceptions impacted how comfortable residents felt accessing services, even when care was geographically or financially available.

Some narratives have historically framed Latino pet owners as hesitant to seek veterinary services, but studies suggest that barriers like cost, language differences, and unfamiliar systems play a larger role in limiting access (5). Assumptions about cultural reluctance to pursue care, especially spay/neuter, are often unfounded and can perpetuate inequities. Without culturally competent care and community engagement, these barriers persist even when services are available (5).

Transportation challenges have been identified as a more significant barrier for pet owners with limited English proficiency (LEP) than for those proficient in English (15). LEP individuals may struggle with accessing reliable transportation, public transit, or ride-sharing services due to language barriers. Many public transit options are reserved for service animals, further limiting options for pet owners (5). Conversations with community members revealed that many families faced the challenge of not having proper supplies—such as carriers, crates, or leashes—to safely transport their pets. This issue was even more pronounced in households with multiple animals, where the lack of equipment made it nearly impossible to bring pets in for care. These challenges contribute to missed veterinary appointments or the inability to seek care altogether (16).

Housing insecurity in Phoenix is high. In 2021, Phoenix was ranked as having the fifth most severe shortage of affordable rental homes of the nation's 50 largest metropolitan areas (17). The U.S. Department of Housing and Urban Development (HUD) defines a “severe rent burden” as spending more than 50% of income on housing (18), meaning many low-income families in Phoenix are left with little financial flexibility for other essential expenses such as food, healthcare, and transportation. In Phoenix, a family of four earning $2,500 per month at the 2023 poverty level would need to allocate nearly 70% of their income to rent a two-bedroom home at fair market value, while a three-bedroom home would consume 95% of their monthly earnings (19). Nationally, research has shown that as housing costs rise, low-income households have less discretionary income, forcing difficult financial trade-offs (20). For pet owners, this often means delaying or forgoing preventive veterinary care, such as vaccinations and parasite prevention, which can lead to more severe health issues and higher costs in the long run. Financial strain may result in pet relinquishment, as owners struggle to afford both housing and pet-related expenses (20).

### 2.2 Details to understand key programmatic elements

#### 2.2.1 Community engagement and needs assessment

In 2022, AAWL hired Community Alliance Consulting to conduct a local bilingual needs assessment, surveying over 600 pet owners in the zip codes nearest the animal shelter (16). Over 73% of respondents identified as Latino, and a significant portion (56.0%) completed the survey in Spanish in zip codes 85006, 85008, 85009, and 85034 (21). This bilingual survey aimed to identify broad trends in access to veterinary care while gathering community input on needs, experiences, and suggestions to inform service planning. Responses highlighted ongoing barriers and emphasized the importance of culturally respectful communication, bilingual access, and affordability (16).

To enhance community participation, the survey was distributed by local Latina promotoras through Unlimited Potential, a local non-profit educational organization. In human healthcare, promotoras have long played a critical role as trusted community members who share knowledge, provide support, and connect families to resources (16, 22). Their involvement has been shown to foster cooperation, strengthen community collaboration, and reduce system failures (21, 22). Recognizing their positive influence in human healthcare, AAWL saw an opportunity to engage with the community on pet issues with the support of the promotoras.

In 2021, as a part of the Alliance for Companion Animals coalition and the Fix.Adopt.Save (FAS) initiative, AAWL analyzed intake and redemption data from the local county animal control. The Alliance for Companion Animals is a local coalition that collaborates with animal welfare groups to increase adoption and foster rates, provide free vaccines, and expand access to spay/neuter services. Their efforts also focus on raising awareness to keep animals with their families and reduce overcrowding in shelters (23). Data collected in 2020 by the coalition helped identify the zip codes 85006, 85008, 85009, and 85034 as key focus areas for the 2022 needs assessment due to their proximity to the AAWL shelter and their high levels of animal intakes and owner surrenders, along with the highest utilization of FAS's free spay/neuter vouchers.

In 2023, AAWL narrowed its focus to a more localized scale with the help of the PetSmart Charities Incubator Program. AAWL chose the 85009 zip code due to its high need per the 2022 needs assessment data and proximity to the shelter. A second survey was sent out to the community through the Institute for Human-Animal Connection (IHAC) within the 85009 zip code (24). The focus was word of word-of-mouth distribution through trusted community networks to create an inclusive and community-engaged approach to gathering input.

Survey responses from 2022 and 2023 revealed consistent barriers to veterinary care in the focus communities, especially cost, language access, and the need for preventative services. In 2022, 55% of respondents (299/543) preferred receiving care in Spanish, underscoring the role of language in access (16). In 2023, over 80% (77/93) said they felt more comfortable with Spanish-speaking providers (24), highlighting that beyond materials, direct communication and cultural rapport matter. Both surveys showed the highest demand for wellness services, vaccines, microchipping, and parasite prevention, while spay/neuter and dental procedures were needed but less urgent. Affordability was a major concern: 44.7% (253/530) reported difficulty paying for basic pet needs, with most only able to afford $11–$25 per visit. Additionally, 59.2% (336/568) had recently needed care but were unable to obtain it. A lack of nearby resources further illustrated gaps in access and communication.

Beyond survey data, AAWL recognized that trust could not be built through one-time interactions. Community members described a long history of “organizaciones fantasmas” or organizations “coming in, promising help, and then disappearing,” which left lasting skepticism. With this in mind, AAWL staff prioritized a consistent presence in neighborhoods, listening before acting, and prioritizing co-created solutions over top-down program design. AAWL translated this philosophy into its clinic operations by embedding practices designed to ensure reliable access, cultural responsiveness, and continuity of care.

#### 2.2.2 Offsite free vaccine clinics

Feedback from both the surveys and direct communication with community members demonstrated the need for low-cost veterinary services delivered in an accessible environment. This led to the development of the off-site clinics conducted in partnership with local organizations and community centers, including Chicanos Por La Causa, a local non-profit organization. These events offered high-volume vaccine services to the public, averaging 70 pets in 3 h, and were operated by a small team of one veterinarian, three technicians, and rotating volunteers. These were performed within the community of Maryvale and provided additional access points for residents who may not have been able to travel to the shelter clinic on weekends.

The offsite clinics were advertised through word of mouth, printed flyers, and social media channels shared by local partners. Bilingual promotoras were invited through community recommendation and were present alongside staff to guide pet owners through services. Their primary contributions included helping to recruit families, guiding them through the process, and serving as familiar, trusted faces that reassured residents about engaging with AAWL services. They also supported the clinics by providing information on the importance of vaccines, recommended vaccine schedules, parasite prevention, and the value of wellness care. Spay/neuter vouchers were distributed on-site to encourage follow-up care and continued engagement. If families declined vouchers, staff and promotoras reinforced the importance of preventative care by sharing information about the Saturday clinic, other low-cost spay/neuter options, and the value of ongoing wellness visits. Families frequently expressed that seeing promotoras, already recognized as reliable community health workers, made them feel more comfortable and willing to participate. This reflects broader findings from human health contexts (21, 22). Promotoras helped bridge the initial gap between AAWL and community members and laid the groundwork for stronger, sustained relationships.

Offering veterinary services in accessible and familiar spaces demonstrated a commitment to understanding local priorities, particularly the need for preventative care and flexible access points. When services were available at the same locations, such as schools and community centers, residents returned for booster vaccines or brought additional pets. Feedback was gathered informally through conversations with attendees, promotoras, and volunteers, as well as staff observations documented during or after the events. Recurring themes, including appreciation for bilingual communication, trust in community-based partners, and difficulty attending clinic hours at the shelter, were discussed by leadership during weekly operations meetings to guide program planning.

With trust as the foundation, this approach was extended into onsite clinic operations, prioritizing consistency, accessibility, and responsiveness to meet community needs and overcome barriers to care.

#### 2.2.3 Onsite clinic operations

In 2023, AAWL's Community Veterinary Clinic (CVC) was operating 5 days a week with once a month vaccine clinics, utilizing contract relief veterinarians and support staff. A financial analysis of this model conducted by AAWL showed that this practice structure was not financially sustainable. On average, only six–nine patients were seen daily. In 2024, AAWL made the strategic decision to switch to a Saturday-only model, operating from 8 a.m. to 6 p.m. This change was informed by survey feedback from both 2022 and 2023, indicating that more people would be able to access services on the weekends.

The Saturday clinic structure consisted of two main components:

- Morning vaccine clinic: from 8 a.m.to 12 p.m., the clinic operated as a high-volume vaccine clinic, where animals received brief exams by a doctor for vaccines and microchipping. Walk-in availability was from 8 a.m.to 9:30 a.m.
- Afternoon wellness appointments: from 12 p.m. to 6 p.m., the clinic offered 20-min wellness appointments for vaccines, wellness exams, and treatment of non-chronic illnesses such as ear infections. These appointments allowed doctors to speak with pet owners and prescribe medications as needed. There were also technician-only appointments for services like nail trims and anal gland expressions.

Owners could sign up independently for appointments through an online sign-up system (SignUpGenius), through the AAWL website, and appointments were manually scheduled into the veterinary software system by an employee during the week.

#### 2.2.4 Staffing and recruitment

**Figure 1:**
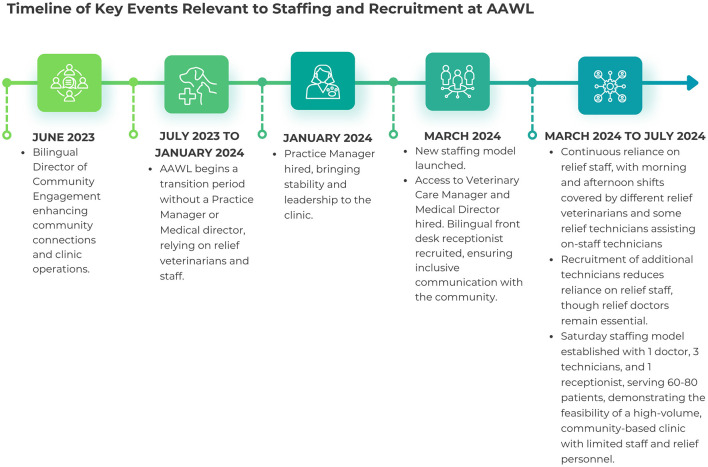
Timeline of key events relevant to staffing and recruitment at AAWL.

AAWL's new clinic model emphasized the importance of bilingual staff to effectively serve the community. In response to staff turnover and survey insights, two key bilingual roles were created: Director of Community Engagement and Access to Vetcare Manager. These roles were crucial in connecting with the Latino community and managing the clinic's operations. The Director handled outreach logistics and partnerships; the Access to Vetcare Manager focused on relationship-building and overseeing off-site services. Figure 1 presents a timeline of key staffing and recruitment events between June 2023 and July 2024, illustrating the clinic's evolving personnel structure during the transition period.

The clinic continued to operate primarily with contract relief medical staff from March 2024 to July 2024. By April 2024, the clinic was able to hire enough technicians as employees to reduce the reliance on contract relief staff, but relief veterinarians remained a key part of the model. A typical Saturday clinic under the new model operated with one veterinarian, three technicians, and one receptionist, comparable staffing to the previous full-service, 5 days a week model but with a higher caseload.

Initially, a lack of clear leadership during clinics led to internal coordination issues, scheduling confusion, and reduced continuity for clients. In response, Community and Medical Leads were designated each week to provide leadership during Saturday clinics, managing volunteers, addressing challenges, and coordinating staff responsibilities. These roles were instrumental in ensuring smooth clinic operations and volunteer satisfaction. The Community Lead managed client check-ins, guiding volunteers, and assisted Spanish-speaking pet owners. The Medical Lead coordinated patient flow, supported staff, and ensured clinical protocols were followed. Both roles were filled by bilingual staff whenever possible to support communication with the majority-Latino community members. Introducing these roles significantly improved staff cohesion and volunteer morale.

During the staffing transition, volunteers expressed a desire for more direct communication and participation. The changes to the clinic model were announced in a February 2024 newsletter, without volunteer input. Volunteers noted the change as abrupt and expressed a desire to have been consulted earlier. In response to this feedback, the CEO held a town hall in March 2024 to acknowledge the concerns, take responsibility for the perceived lack of transparency, and reinforce the importance of the volunteer community. Volunteers expressed interest in more interactive communication, such as in-person meetings, advance notice of changes, and opportunities for input. AAWL reaffirmed its commitment to communication and trust-building by involving volunteers in outreach, clinic observation, and operational feedback throughout March-July 2024. These efforts supported continuity and strengthened engagement during the transition.

The shift to a Saturday-only model represented a rapid organizational pivot, prompted by the unexpected departure of the clinic's license-holding veterinarian in early 2024. This required immediate restructuring to comply with veterinary regulations. Beyond logistical changes, the transition required a broader culture shift, from slower-paced weekday appointments to a fast-paced, high-volume Saturday wellness model. Staff had to adapt to new roles, workflows, and expectations.

#### 2.2.5 Transition and engagement outcomes

The new clinic model opened in March of 2024, and implementation was documented through July 2024. Under the new Saturday-only model, the clinic served as many or more patients in a single day than it previously saw in an entire week. The old model, operating Monday–Friday, averaged 6–9 patients per day, or 36–54 animals weekly. In contrast, the new model saw an average of 55 patients per Saturday in March, increasing to 70 patients per Saturday in April, and averaging 66 patients per Saturday by July. Consolidating services into a single high-volume day allowed the clinic to maintain, if not exceed, its previous capacity while improving operational efficiency and resource allocation since it did not require hiring contract relief veterinarians and staff for five additional days. To meet the needs of the Latino community served, AAWL also prioritized hiring bilingual front desk staff and ensured bilingual support staff and volunteers were present each week to assist with client check-in, answer questions in Spanish, and help families navigate clinic flow upon arrival.

Despite these successes, the operation faced a significant challenge with no-show appointments, which consistently impacted the clinic's efficiency and resource allocation. The no-shows not only led to unused appointment slots but also created inefficiencies in managing the high demand for services. Patients who could have been accommodated were often placed on waitlists due to full bookings, only for those slots to go unfilled due to no-shows.

To address this, the clinic team introduced walk-in appointments from 8:00 to 9:30 a.m. starting in May 2024, specifically targeting the time frame when the highest rate of no-shows was typically observed. This allowed the clinic to provide an opportunity for those unable to book appointments to receive care. The walk-in slots were limited to 15 patients. This was communicated through bilingual flyers, signage at the clinic, and updated website information. Once capacity was reached, clients were offered the option to join a waitlist, return the following Saturday, or, sometimes able to be seen the same day if willing to have a longer wait time. Although labeled “walk-in,” these slots were often filled quickly, leading to clients arriving very early in the morning to secure their slot, suggesting high demand and limited alternatives. While the exact reasons for this pattern cannot be fully determined, many of the population being served have a clear need for flexibility, as families often face unpredictable work schedules, transportation barriers, and financial constraints.

AAWL took additional steps to better understand and address the reasons behind the high no-show rates. Improved communication strategies, such as sending appointment reminders and providing clearer instructions on what to expect during visits, were considered and trialed to reduce no-shows to varying effects. In addition, a small $5 deposit was introduced as a pilot strategy to encourage attendance and help offset the impact of missed appointments.

In response to Arizona's extreme summer heat, AAWL adjusted the clinic flow to allow pets and owners to wait indoors or in their vehicles, and provided water. The original setup had clients waiting outdoors in the sun or crowding in the small reception area. The new flow staggered appointments and allowed for safer, more comfortable wait options. These adjustments were well-received and continued beyond the summer months to help with capacity and wait times.

Communication improvements, including the hiring of an additional receptionist, helped to address the backlog of voicemail and enhance client satisfaction. Prior to these changes, staff reported that clients expressed frustration with delayed responses to voicemail, unclear instructions about appointment logistics, and inconsistent information provided at check-in. Satisfaction concerns were identified through a combination of volunteer observations, informal staff debriefs, and direct client communications. In response, AAWL implemented several communication-focused improvements. The newly hired receptionist helped reduce the voicemail backlog, answered client questions during the week, and provided more consistent instructions ahead of appointments. These efforts contributed to a more streamlined experience for clients and reduced confusion on clinic days.

Informal feedback from both clients and volunteers also reflected increased trust and satisfaction with the new model. Many expressed appreciation for the bilingual services, improved communication, and the effort to create a more inclusive and responsive clinic environment.

## 3 Discussion

This case study highlights the importance of culturally responsive, community-based care that adapts to local needs rather than assuming uniformity across underserved populations. The lessons learned here may be useful to other organizations serving Latino communities or implementing similar wellness-focused clinic models, but these efforts must always be grounded in population-specific contexts.

By engaging directly and listening to the community's concerns, AAWL was able to create a program that not only built strong relationships but also utilized funds and resources more efficiently. The key leaders in this initiative regularly reassessed the changes to ensure a sustainable model. Leadership met weekly during implementation to review clinic flow, appointment demand, and client feedback, using a continuous improvement model similar to those seen in other community health collaborations (4). This continual review process shows that community-based programs are not static but require ongoing evaluations to remain impactful.

Several key lessons emerged from the clinic's first few months under the new model. First, enhancing communication strategies, both internal and external, is essential to maintaining staff morale and client satisfaction. Bilingual service expansion and clearer client instructions helped address common barriers related to language access and clinic navigation, consistent with communication strategies reported in literature as essential to reaching diverse populations (4, 25). Beyond language, it became equally important to have staff who were not only bilingual but also from the community itself and able to relate to clients' lived experiences. For example, AAWL's Access to Vet Care Manager was both bilingual and a trusted face in the community, having built relationships through prior outreach. This individual became a critical point of contact for families as they navigated complicated systems to receive support. Distributing educational materials that covered topics like vaccine schedules, flea/tick prevention, and spay/neuter options further strengthened the clinic's outreach to the local community, especially when paired with staff who were trusted community members able to provide navigation support and build ongoing relationships.

Initially, there was a high no-show rate for scheduled appointments at the on-site clinic. Efforts to reduce this included more robust appointment reminders and offering flexible scheduling options in the form of walk-in slots. The demand for these slots may be an indication that many pet owners in this community face challenges and require flexibility in appointments, and walk-ins can provide that. Building in an option for flexibility in scheduling could improve operational efficiency in clinics in similar communities.

The cross-training initiative for veterinary support staff not only strengthened shelter and clinic operations but also enhanced the staff's ability to serve the community more effectively. Hands-on learning, particularly for entry-level staff, is aligned with broader workforce development strategies in animal welfare and human healthcare that emphasize upskilling and internal mobility as ways to build resilience and retention (26). Similarly, volunteer engagement was reframed as a form of community partnership. By introducing structured orientation programs, feedback mechanisms, and assigning a weekly community lead, AAWL reinforced volunteers' role as cultural ambassadors who helped clients feel welcome and supported. This approach reflects findings from other high-volume care models, where structured onboarding not only increases retention but also strengthens trust between organizations and the communities they serve (25).

The internal transition required a rapid organizational pivot and led to a culture shift that reshaped both staff and volunteer dynamics. Moving from slower-paced, individualized weekday appointments to high-volume Saturday wellness care demanded new ways of working and challenged existing expectations. While these shifts were operational in nature, they also had implications for community engagement: staff and volunteers who felt uncertain or excluded from the process risked transmitting that hesitancy to clients. Securing buy-in from these key stakeholders became essential, underscoring how internal culture and external trust are interconnected (27). For future change efforts, continued strong communication and inclusive decision-making will not only improve efficiency and team cohesion but also reinforce the credibility of community-facing services.

The clinic has relied heavily on a variety of revenue streams to support its operations. While private-paying clients have provided a primary source of revenue, a significant portion of the clients, particularly those from the targeted zip codes (85006, 85008, 85009, and 85034), have been subsidized by grant funds from partners like the Alliance for Companion Animals and PetSmart Charities. These grants have been essential in making services accessible to those in need; however, they are not a stable or permanent solution. Grant funding can be inconsistent and often fluctuates with changing funder priorities, making it an unreliable long-term strategy. To address this, AAWL acknowledges the need to incorporate additional strategies, such as implementing low-cost deposits, offering self-pay options for certain medications, such as flea/tick preventatives and basic antibiotics, and introducing no-interest payment plans. These alternatives have shown promise in both human and veterinary healthcare contexts as strategies to reduce financial drop-offs in care, instances where clients delay or decline recommended treatments due to cost (28, 29). These measures aim to reduce the clinic's dependence on grants and create a more predictable revenue stream, ensuring the clinic can continue to meet the community's needs while maintaining financial viability.

Consistent clinic attendance and informal feedback from clients and promotoras suggest unmet demand for expanded veterinary care access, including surgical procedures. While this case study focused on preventative and wellness services, AAWL is evaluating when to expand surgical offerings such as spay/neuter and dentals to meet this demand. While attendance data was disaggregated by zip code, the number of pet owners in need of spay/neuter services was not formally measured; however, community members frequently expressed interest in surgical services. If supported, additional funding, staffing, and mobile resources would be required, and those considerations are in discussion.

Lessons from community discussions extended beyond clinical services. Residents consistently identified preventative care—vaccines, parasite prevention, and spay/neuter—as their highest priorities, while also emphasizing that affordability was a constant barrier. Families described a history of organizations “coming in and disappearing,” which reinforced that consistency and reliability were as important as affordability in building trust. Community members also highlighted structural challenges, such as the lack of sidewalks, poor lighting, and limited green spaces, which constrained their ability to walk pets safely or socialize them (16, 24). Together, these insights highlighted that true access to veterinary care must consider the broader social and environmental conditions in which families live, not just the cost of medical services.

Community members consistently emphasized that promotoras are their first point of contact for health information. The use of promotoras at AAWL aligns with models in human healthcare. While promotoras in typical healthcare settings focus on chronic disease prevention and management, AAWL's adaptation emphasized logistical support, cultural translation, and education on preventative veterinary care. This reflects the flexibility of promotora models and underscores the value of leveraging community health strategies in animal welfare contexts. AAWL is developing a pilot for delivering pet care and health resources via a promotora model. The curriculum is being shaped with promotora and community feedback to ensure it is culturally responsive and community-owned. This process illustrates that community voices should not be an afterthought but rather the foundation for designing sustainable veterinary care models. The authors believe that the promotora curriculum and the use of promotoras in access to veterinary care holds significant potential for adaptation to other settings, as it is grounded in community-identified needs and delivered through trusted messengers, strengthening veterinary access by ensuring knowledge lives within the community itself.

The most critical lesson learned is that expanding access to veterinary care is not only about delivering more services, but about understanding how communities themselves want to engage. For funders and organizations, the lesson is clear: expanding access to care is not simply about building clinics, but about co-creating systems of trust and resilience with the very people they are meant to serve, and pivoting as necessary, when structures are not serving their intended purpose. By listening deeply to community voices, honoring local practices, and designing processes that reflect daily realities, organizations can build approaches rooted in trust and resilience. When systems are built with—not just for—communities, care becomes not only more accessible, but also sustainable and embraced.

### 3.1 Acknowledgment of conceptual or methodological constraints

Cultural bias may be present from the authors who are engaging in this work and have relationships within the community studied. These relationships include prior or current roles within the organization and direct involvement with the clinic transition process.

## Data Availability

The original contributions presented in the study are included in the article/supplementary material, further inquiries can be directed to the corresponding author.

## References

[1] King E Mueller M Wolfus G McCobb E. Assessing Service-learning in community-based veterinary medicine as a pedagogical approach to promoting student confidence in addressing access to veterinary care. Front Vet Sci. (2021) 8:644556. 10.3389/fvets.2021.644556 34222392 PMC8245678

[2] Kogan LR Accornero VH Gelb E Slater MR. Community veterinary medicine programs: pet owners' perceptions and experiences. Front Vet Sci. (2021) 8:678595. 10.3389/fvets.2021.678595 34169110 PMC8217603

[3] LaVallee E Mueller MK McCobb E. A systematic review of the literature addressing veterinary care for underserved communities. J Appl Anim Welf Sci. (2017) 20:381–94. 10.1080/10888705.2017.1337515 28657796

[4] Pasteur K Diana A Yatcilla JK Barnard S Croney CC. Access to veterinary care: evaluating working definitions, barriers, and implications for animal welfare. Front Vet Scince. (2024) 11:1335410. 10.3389/fvets.2024.1335410 38304544 PMC10830634

[5] Decker Sparks JL Camacho B Tedeschi P Morris KN. Race and ethnicity are not primary determinants in utilizing veterinary services in underserved communities in the United States. J Appl Anim Welf Sci. (2018) 21:120–9. 10.1080/10888705.2017.1378578 28960091

[6] Baker T Rock M Bondo K Van Der Meer F Kutz S. 11 years of regular access to subsidized veterinary services is associated with improved dog health and welfare in remote northern communities. Prev Vet Med. (2021) 196:105471. 10.1016/j.prevetmed.2021.105471 34509773

[7] Blackwell MJ O'Reilly A. Access to veterinary care–a national family crisis and case for one health. Adv Small Anim Care. (2023) 4:145–57. 10.1016/j.yasa.2023.05.003

[8] Frank JM Carlisle-Frank PL. Analysis of programs to reduce overpopulation of companion animals: do adoption and low-cost spay/neuter programs merely cause substitution of sources? Ecol Econ. (2007) 62:740–6. 10.1016/j.ecolecon.2006.09.011

[9] Mueller MK Chubb S Wolfus G McCobb E. Assessment of canine health and preventative care outcomes of a community medicine program. Prev Vet Med. (2018) 157:44–9. 10.1016/j.prevetmed.2018.05.016 30086848 PMC6397959

[10] U.S. Census Bureau. Phoenix city, Arizona - Census Bureau Profile. Available online at: https://data.census.gov/profile/Phoenix_city,_Arizona?g=160XX00US0455000#populations-and-people (Accessed August 15, 2024).

[11] City of Phoenix Planning and Development Department. Central City Village Character Plan Phoenix, AZ: City of Phoenix. Available online at: https://www.phoenix.gov/content/dam/phoenix/pddsite/villagessite/documents/central%20city%20village%20character%20plan.pdf (Accessed February 14, 2025).

[12] Oreopoulos P Salvanes KG. Priceless: the nonpecuniary benefits of schooling. J Econ Perspect. (2011) 25:159–84. 10.1257/jep.25.1.159

[13] Flores G. Language barriers to health care in the United States. N Engl J Med. (2006) 355:229–31. 10.1056/NEJMp058316 16855260

[14] Coe JB Adams CL Bonnett BN. A focus group study of veterinarians' and pet owners' perceptions of veterinarian-client communication in companion animal practice. J Am Vet Med Assoc. (2008) 233:1072–80. 10.2460/javma.233.7.1072 18828715

[15] Landau RE Beck A Glickman LT Litster A Widmar NJO Moore GE. Use of veterinary services by Latino dog and cat owners with various degrees of English-language proficiency. J Am Vet Med Assoc. (2016) 248:681–9. 10.2460/javma.248.6.681 26953923

[16] Arizona Animal Welfare League. AAWL Community Survey: November 2022 Final Report. Phoenix, AZ: Arizona Animal Welfare League (2022). Available online at: https://aawl.org/sites/default/files/aawl-cac_community_survey_nov_2022_final.pdf (Accessed March 28, 2025).

[17] National Low Income Housing Coalition. T*he Gap: A Shortage of Affordable Homes*. Washington, DC: National Low Income Housing Coalition (NLIHC) (2024).

[18] Dawkins C Sik Jeon J. Rent Burden in the Housing Choice Voucher Program. Washington, DC: U.S. Department of Housing and Urban Development, Office of Policy Development and Research (2017).

[19] Burns & Associates Inc., a division of Health Management. 2023 Community Assessment 1-Year Update: Report and Appendices. Phoenix, AZ: City of Phoenix Human Services Department (2023). Available online at: https://www.phoenix.gov/content/dam/phoenix/humanservicessite/documents/hsd-/2023%20cna%20community%20assessment%20report%201-year%20update%20-%20report%20and%20appendices.pdf (Accessed February 22, 2025).

[20] Bhutta N McGranahan L Ringo D. Assessing the Severity of Rent Burden on Low-Income Families. Available online at: https://www.federalreserve.gov/econres/notes/feds-notes/assessing-the-severity-of-rent-burden-on-low-income-families-20171222.html (Accessed February 22, 2025).

[21] Cain CL Orionzi D O'Brien M Trahan L. The power of community voices for enhancing community health needs assessments. Health Promot Pract. (2017) 18:437–43. 10.1177/1524839916634404 27091607

[22] Schwingel A Wiley AR Teran-Garcia M McCaffrey J Gálvez P Vizcarra M. *Promotoras* and the semantic gap between Latino Community Health Researchers and Latino Communities. Health Promot Pract. (2017) 18:444–53. 10.1177/1524839916670576 27760810

[23] Fixadoptsave. Fix.Adopt.Save:About. Available online at: https://www.fixadoptsave.org/about (Accessed August 28, 2024).

[24] Arizona Animal Welfare League. Arizona Animal Welfare League (AAWL) – Final Data Report June-October 2023. Phoenix, AZ: Arizona Animal Welfare League (2023). Available online at: https://aawl.org/sites/default/files/eng_-_sp_arizona_animal_welfare_league_ihac_pc_phase_1_final_data_report_2023.pdf (Accessed March 28, 2025).

[25] Niemiec R Champine V Frey D Lobdell A Steele A Vaiden C . Veterinary and pet owner perspectives on addressing access to veterinary care and workforce challenges. Front Vet Sci. (2024) 11:1419295. 10.3389/fvets.2024.1419295 39086761 PMC11289980

[26] Smith SM George Z Duncan CG Frey DM. Opportunities for expanding access to veterinary care: lessons from COVID-19. Front Vet Sci. (2022) 9:804794. 10.3389/fvets.2022.804794 35478604 PMC9036088

[27] Mauck CR Vincent MR Robertson JV. Unlocking collaborative dynamics: exploring veterinarian-leadership relationships in animal shelters. J Shelter Med Community Anim Health. (2025) 4:108. 10.56771/jsmcah.v4.108

[28] Kipperman BS Kass PH Rishniw M. Factors that influence small animal veterinarians' opinions and actions regarding cost of care and effects of economic limitations on patient care and outcome and professional career satisfaction and burnout. J Am Vet Med Assoc. (2017) 250:785–94. 10.2460/javma.250.7.785 28306486

[29] Bir C Ortez M Olynk Widmar NJ Wolf CA Hansen C Ouedraogo FB. Familiarity and use of veterinary services by U.S. resident dog and cat owners. Animals. (2020) 10:483. 10.3390/ani10030483 32183120 PMC7143178

[30] Bureau of Women's and Children's Health Arizona Department of Health Services. Maryvale Village Primary Care Area. Phoenix, AZ: Arizona Department of Health Servicesd (2022).

[31] City of Phoenix Human Services Department. 2024 HSD Community Assessment Report Year 2 Update and Appendices. Phoenix, AZ: City of Phoenix Human Services Department (2024).

[32] City of Phoenix Planning and Development Department. Central City Primary Care Area (PCA). Phoenix, AZ: City of Phoenix (2021).

